# Supplementary material for: Novel Structures of Type 1 Glyceraldehyde-3-phosphate Dehydrogenase from Escherichia coli Provide New Insights into the Mechanism of Generation of 1,3-Bisphosphoglyceric Acid
Source: Biomolecules. 2021 Oct 22;11(11):1565. doi: 10.3390/biom11111565 (PMC8615399; doi:10.3390/biom11111565)
Supplement: Supplementary file 1 [file biomolecules-11-01565-s001.zip › biomolecules-1365426-supplementary.pdf]

Crystal Structure of Type1 Glyceraldehyde-3-Phosphate Dehydrogenase from *Escherichia coli* Provides New Insight into BPG Generation and Catalytic Mechanism

Li Zhang[1], Meiruo Liu[1], Luyao Bao[1], Kristina I. Boström[2], Yucheng Yao[2], Jixi Li[1], Shaohua Gu[1] and Chaoneng Ji[1, 3*]

[1] State Key Laboratory of Genetic Engineering, School of Life Sciences, Fudan University, Shanghai, 200438, People's Republic of China Li Zhang email: lizhang1@g.ucla.edu; Meiruo Liu email: mandymrliu@gmail.com, Luyao Bao email: baoly@fudan.edu.cn Jixi Li email: lijixi@fudan.edu.cn Shaohua Gu shaohuagu@fudan.edu.cn

[2] Division of Cardiology, David Geffen School of Medicine at UCLA, Los Angeles, CA, 90095-1679, USA Kristina I. Boström email: kbostrom@mednet.ucla.edu Yucheng Yao email: yyao@mednet.ucla.edu

[3] Shanghai Engineering Research Center of Industrial Microorganisms, Shanghai, 200438, People's Republic of China

* Correspondence e-mail: chnji@fudan.edu.cn

Supplementary Materials:

Table S1 Sequence of mutants' primers

Primer name	Primer oligo 5'-3'
C150S-F	CCATTGTTTCCGTGGCGTCATCAACCACTAACTGTCTTG
C150S-R	CAAGACAGTTAGTGGTTGATGACGCCACGGAAACAATGG
C150A-F	GACACCATTGTTTCCGTGGCGTCAGCAACCACTAACTGTC
C150A-R	GACAGTTAGTGGTTGCTGACGCCACGGAAACAATGGTGTC
H177A-F	CACGATGACGACCATTGCAGCCTATACTGGCACCCAGTC
H177A-R	GACTGGGTGCCAGTATAGGCTGCAATGGTCGTCATCGTG

Table S2 The composition of the precipitant solutions used to obtain protein crystals

Crystal name	Crystallization conditions
WT.NAD	100 mM sodium acetate pH4.6, 30%(w/v) PEG400, 200 mM $Ca(OAC)_2$
C150S.NAD	100 mM sodium acetate pH4.5, 30% (w/v) PEG400, 200 *mM* $Ca(OAC)_2$
C150A.NAD	100 mM sodium Cacodylate pH 6.3, 18% (w/v) PEG1000, 200 mM $MgCl_2$
H177A.NAD	100 *mM* sodium cacodylate pH 6.5, 18%(w/v) PEG1000, 200 mM $MgCl_2$
C150A+H177A.NAD	100 *mM* sodium cacodylate pH 6.3, 20%(w/v) PEG1000, 200 mM $MgCl_2$
WT.NAD.PO_4	100 mM sodium PBS pH 6.1, 16%(w/v) PEG1000, 200 mM $MgCl_2$
C150A.NAD.PO_4	100 mM sodium cacodylate pH 6.3, 20%(w/v) PEG1000, 200 mM $MgCl_2$
C150S.NAD.PO_4	100 mM sodium cacodylate pH 6.3, 20%(w/v) PEG1000, 200 mM $MgCl_2$
WT.NAD.G3P	100 mM sodium PBS pH 6.1, 16%(w/v) PEG1000, 200 mM $MgCl_2$
C150S. NAD.G3P	100 mM sodium PBS pH 6.1, 16%(w/v) PEG1000, 200 mM $MgCl_2$
C150A+H177A.G3P	100 mM sodium PBS pH 6.6, 16%(w/v) PEG1000, 200 mM $MgCl_2$
Thioacyl intermediate	100 mM sodium PBS pH 6.6, 16%(w/v) PEG1000, 200 mM $MgCl_2$
C150S.NAD.BPG	100 mM sodium PBS pH 6.2, 16%(w/v) PEG1000, 200 mM $MgCl_2$
H177A.NAD.BPG	100 mM sodium PBS pH 6.6, 16%(w/v) PEG1000, 200 mM $MgCl_2$

Table S3 X - ray data collection and refinement statistics

	Holoenzyme				Ternary complex	
	C150S.NAD	C150A.NAD	H177A.NAD	C150A+H177A.NAD	C150S.NAD.PO_4	C150A.NAD.PO_4
Data collection						
PDB code	7C5L	7C5J	7C5O	7C5N	7C5G	7C5I
Wavelength(Å)	0.9776	0.97776	0.97776	0.97776	0.97776	0.97776
Resolution range(Å)	50-2.1 (2.14-2.1)[a]	50-1.98 (2.01-1.98)	50-1.98 (2.01-1.98)	50-1.99 (2.02-1.99)	50-1.98 (2.01-1.98)	50-2.49 (2.53-2.49)
Space group				$P4_12_12$		
Unit-cell paramters (Å, °)	a=b=89.40, c=340.266, α=β=γ=90	a=b=90.0, c=340.867, α=β=γ=90	a=b=89.97, c=342.25, α=β=γ=90	a=b=89.376, c=341.506, α=β=γ=90	a=b=90.124, c=340.648, α=β=γ=90	a=b=89.679, c=341.261, α=β=γ=90

Completeness (%)	100(100)	90.9(87.2)	99.1(97.4)	100(100)	90.8(83.6)	100 (100)
R_{merge} (%)	17.4(54.9)	17(56.5)	17.9(48.9)	20.8(88)	17.1(55.9)	19.6(77.6)
Mean I/δ	19.7(4.33)	10.9(2.5)	21.3(6.75)	18.5(5.25)	13.6(3.4)	12(4)
No. unique reflections	82932	89856	98318	96080	90257	50448
Redundancy	19.4	8.7	20	19.5	13.6	19.2
Refinment						
Resolution range(Å)	47.89-2.09 (2.17-2.09)	48.05-1.98 (2.05-1.98)	43.51-1.98 (2.05-1.98)	44.69-1.99 (2.06-1.99)	44.67-1.98 (2.05-1.98)	48.03-2.48 (2.57-2.48)
Rwork/Rfree (%)	15.62/19.97	15.47/20.14	15.7/19.86	16.71/21.77	15.46/20.26	16.74/22.31
Wilson B-factor (Å^2)	21.4	18.7	24	24.7	18.86	32.08
average B factor(Å^2)	23.87	20.7	26.31	27.3	20.21	33.42
B factor(Å^2)						
Protein	23.45	20.19	25.66	26.48	19.75	33.68
Water	28.69	25.98	31.76	33.58	25.22	27.98
NAD^+, PO_4, G3P, BPG	21.62	17.9	30.03	34.18	18.32	35.21
No. NAD/PO4/G3P/BPG	4/0/0/0	4/0/0/0	4/0/0/0	4/0/0/0	4/5/0/0	4/8/0/0
No. water molecules	987	1072	990	1104	995	544
Root mean square deviation						
(Bond lengths(Å)	0.017	0.017	0.02	0.019	0.031	0.042
Bond angles(deg)	1.82	1.8	1.93	1.86	1.82	1.62
Ramachandran Plot (%)						
favored	97	97	97	96	96	95
outliers	0.075	0	0	0	0	0.075
Rotamer outliers (%)	0	1	0.91	0.075	1.3	0.73
Clash core	2.31	1.45	1.89	2.17	2.02	1.88

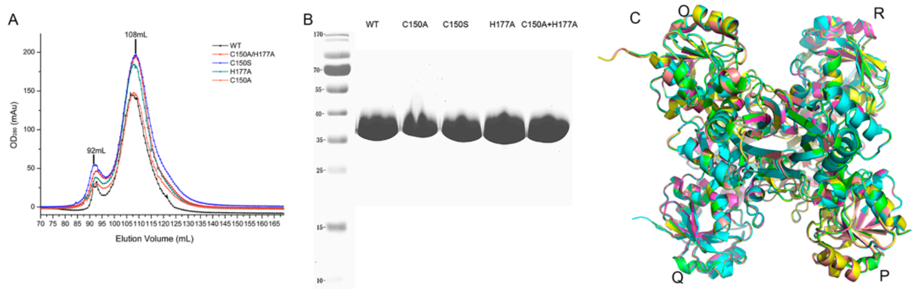

Figure S1 Purification and enzyme characteristics of *Ec*GAPDH1 mutants A. Gel filtration profile of *Ec*GAPDH1 mutants on a Hip Trep@26/60 sephacryl S-100 high resolution column. All *Ec*GAPDH1 mutants were eluted at a 108 ml peak. B. After Ni-NTA affinity chromatography and gel-filtration chromatography, *Ec*GAPDH1 mutants were loaded on a 12% SDS-PAGE gel. C. C150S.NAD, C150A.NAD, H177A.NAD, C150A+177A.NA align to WT.NAD.

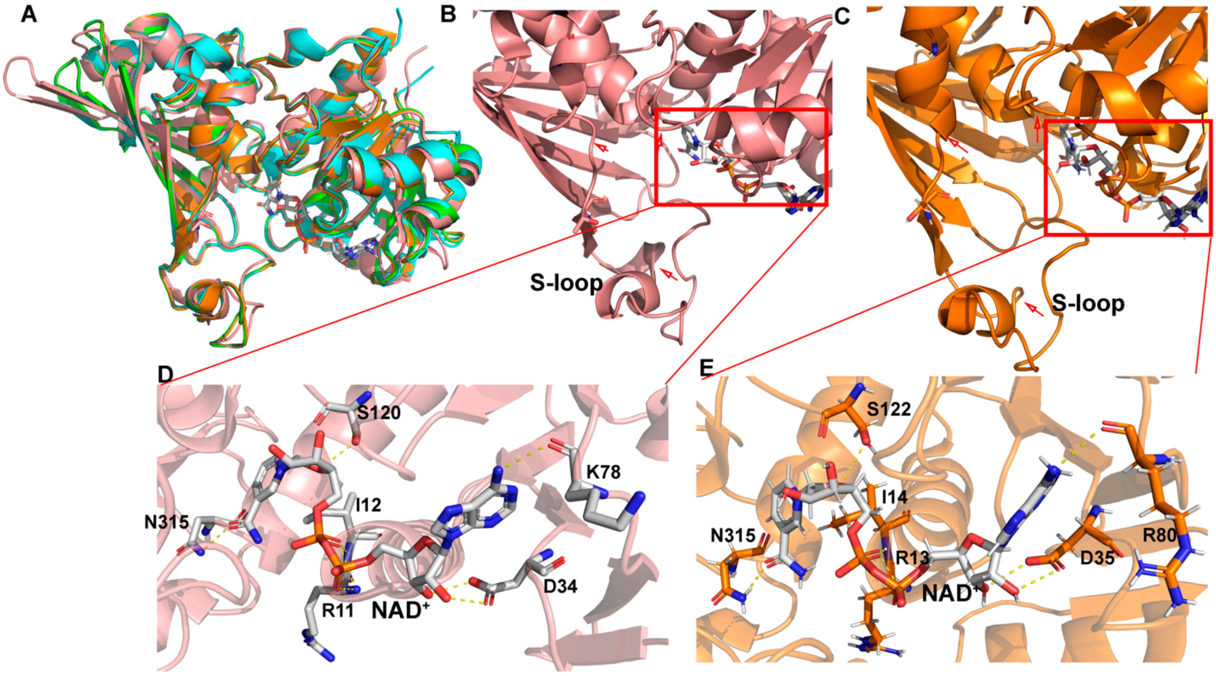

Figure S2. NAD^+ in EcGAPDH1 and GAPDH from Homo sapiens. A. Align structures of O subunit of C150S.NAD (PDB code: 7C5L, wheat), and GAPDH from homo sapiens (PDB code: 4WNC, orange)[1], Human testis (PDB code: 5C7L, cyan)[2], human liver (PDB code: 1U8F, green)[3]. B-C O subunit of C150S.NAD and GAPDH from homo sapiens (PDB code: 4WNC). D, E are the NAD^+ binding forms of structure B and C.

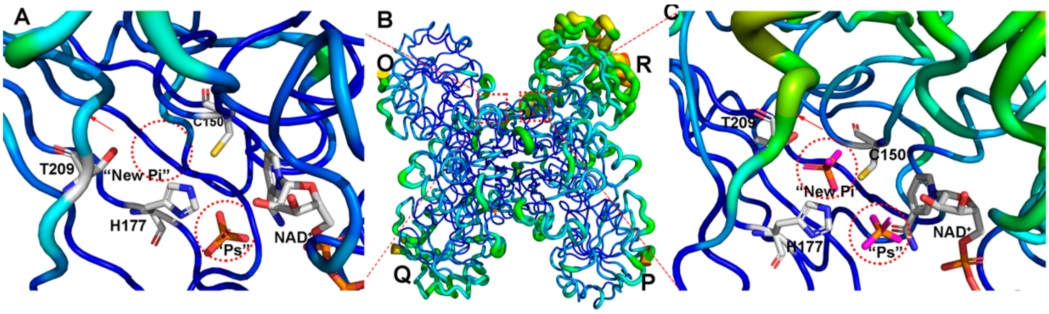

Figure S3 B factor distribution in WT.NAD.PO_4. A, C from O and R subunit of WT.NAD.PO_4, respectively. The wider and redder tubing indicates a higher B-factor. The different part was marker use red circle and arrow.

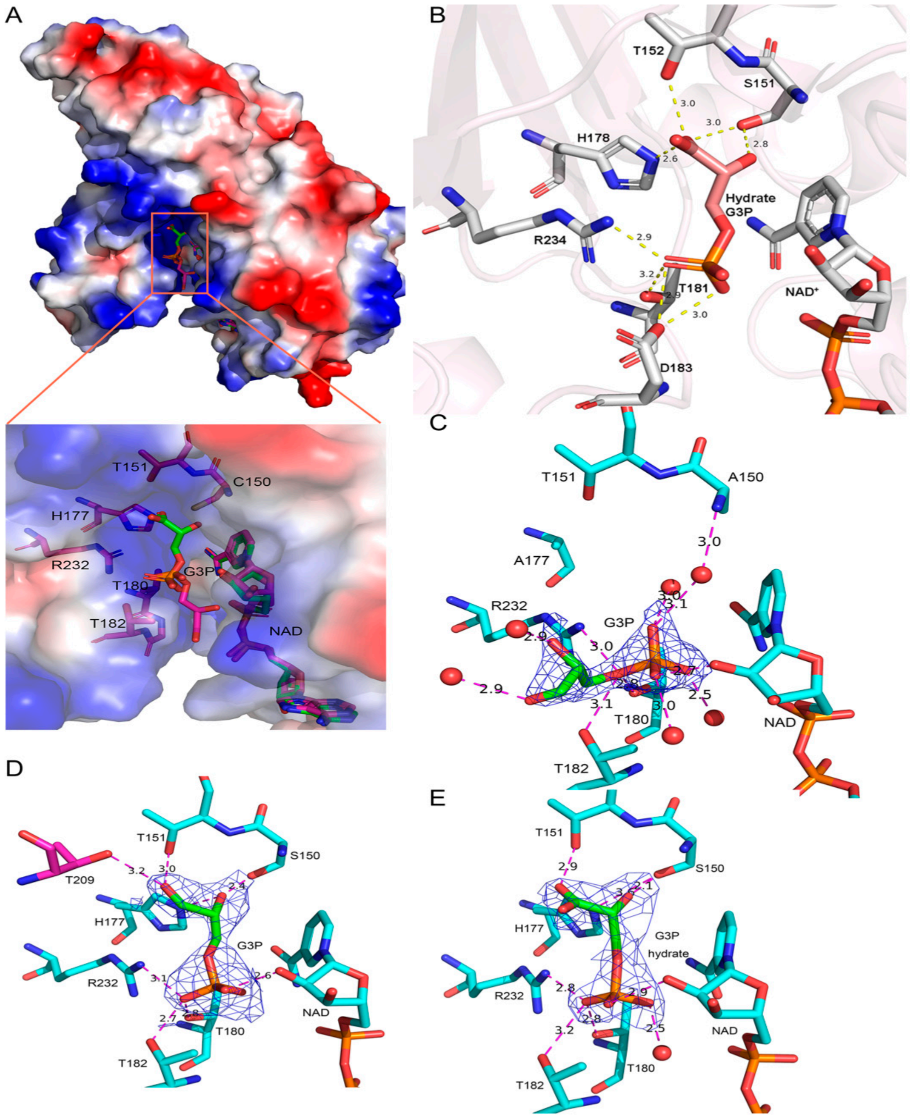

Figure S4 The different binding forms of G3P. A. The R subunit of WT.NAD.G3P is depicted as electrostatic surface potential. Blue represents a positive potential and red depicts a negative potential. Superimposition of the R subunit of *Tm*GAPDH (3KV3) and WT.NAD.G3P shows G3P in opposing structures. G3P is shown to exist in a positively charged pocket. The top panel shows G3P as a monomer. The bottom panel shows the amino acids and NAD^+ interacting with G3P. B. The R subunit of *Tm*GAPDH. G3P is in a gem-diol form (hydrate form). C. A stereo-view of simulated annealing (2mFo-DFc) omit map of G3P; the map contoured at 1.0 σ in the Q subunit of C150A+H177A.NAD.G3P. The polar interactions (shown in magenta) of G3P with water molecules, NAD^+, and amino acid residues are highlighted. The O, Q subunit of C150S.NAD.G3P show same express way with Q subunit of C150A+H177A.NAD.G3P. D. C150S.NAD.G3P (Chain O). E. C150S.NAD.G3P (Chain Q); G3P is in hydrate form. C, D. G3P is in aldehyde form. E. Superimposition of G3P as mentioned above. G3P molecules are shown as sticks and the subunit is the surface.

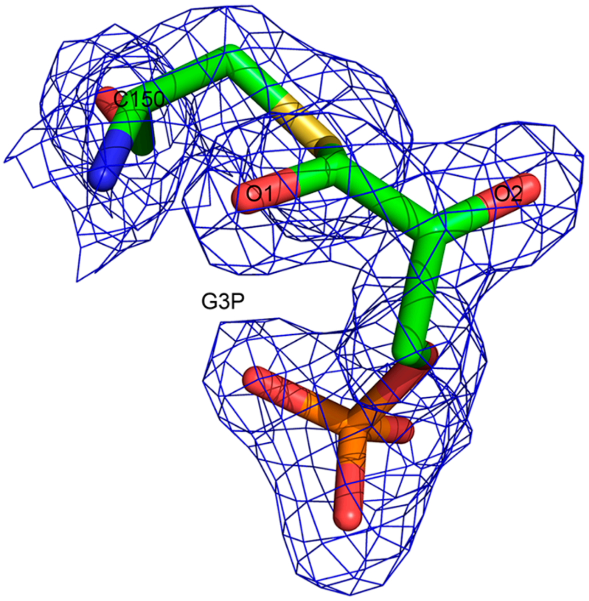

Figure S5 Structure of thioacyl intermediate. Stereo-view of simulated annealing omit (2Fo−DFc) map of the thioacyl intermediate contoured at 1.0 σ.

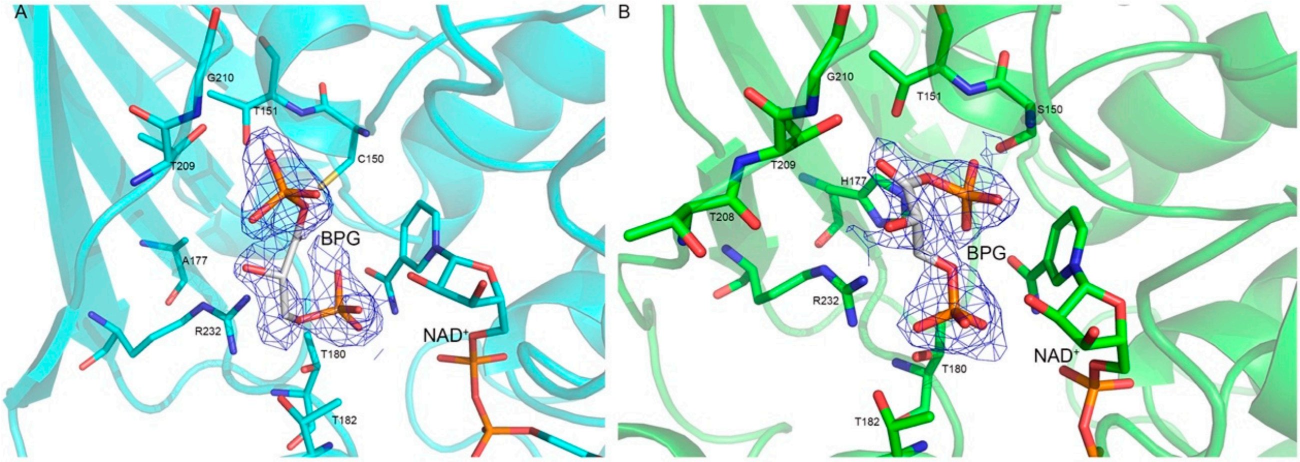

Figure S6 Structure of GAPDH binding BPG. A-B. Stereo-view of simulated annealing omit (2Fo−DFc) map of the P subunit of H177A.NAD.BPG and O subunit of C150S.NAD.BPG contoured at 0.9 sigma. Cartoon representation of the overall structure is shown. The amino acids that interact with BPG are shown as sticks.
